# Enhanced Fluorescence of Near-Infrared Anti-CEA Antibodies for Visualizing Colorectal Cancers Using Modified Heptamethine Cyanines

**DOI:** 10.1007/s11307-025-02076-3

**Published:** 2026-02-10

**Authors:** K. Cox, J. Jitender, S. Mehta, Dong-Hao Li, S. Amirfakhri, R. M. Hoffman, J. Shively, P. Yazaki, M. J. Schnermann, M. Bouvet, T. M. Lwin

**Affiliations:** 1 Department of Surgery, University of California San Diego, San Diego, CA USA; 2 VA San Diego Healthcare System, San Diego, CA USA; 3 Department of Immunology & Theranostics, Beckman Research Institute, Duarte, CA USA; 4 Chemical Biology Laboratory, Center for Cancer Research, National Cancer Institute, Bethesda, MD USA; 5 Department of Surgery, City of Hope National Medical Center, Duarte, CA USA

**Keywords:** Fluorescence-guided surgery, Molecular imaging, Near-infrared fluorescent dyes, Anti- carcinoembryonic antigen antibody, Heptamethine cyanine dyes

## Abstract

**Background:**

The clinical success of fluorescence-guided surgery is dependent on tumor-specific probes that can achieve high tumor-to-background contrast. Conventional near-infrared (NIR) fluorophores often alter the pharmacokinetic properties of the parental molecule that result in aggregation, altered biodistribution, and impaired tumor targeting.

**Methods:**

This present study evaluates two charge-balanced heptamethine cyanine dyes, FNIR-Tag-766 and FNIR-Tag-804, conjugated to a humanized anti-carcinoembryonic antigen antibody (M5A). Their performance was compared to the standard M5A-IR800CW conjugate in both subcutaneous and orthotopic colorectal cancer xenograft models in mice. Mean fluorescence intensity (MFI), tumor-to-background ratio (TBR), and ex vivo biodistribution were compared.

**Results:**

The M5A-FNIR-766 conjugate produced a greater MFI in tumors at all timepoints compared to M5A-IR800CW, resulting in a significantly improved TBR. Ex vivo analysis at 96 h confirmed higher tumor accumulation for M5A-FNIR-766 and revealed a reduction in hepatic signal for both M5A-FNIR-Tag conjugates. The observations were concordant in the clinically relevant orthotopic model.

**Conclusion:**

Antibody-fluorophore conjugates linked to the charge-modified FNIR-Tag dyes provide improved tumor-specific signals and a more favorable biodistribution profile than the same antibody conjugated to a conventional dye. By achieving superior performance through intrinsic fluorophore design rather than complex bioconjugation strategies, the approach provides a clinically translatable advancement for tumor-specific FGS.

**Supplementary Information:**

The online version contains supplementary material available at 10.1007/s11307-025-02076-3.

## Introduction

Advances in molecular biology and protein engineering have facilitated the development of probes that can be linked to fluorophores for fluorescent intra-operative tumor visualization and fluorescence-guided surgery (FGS) [1]. Among these, the use of monoclonal antibodies (mAb’s) as fluorescent tracers have demonstrated promise since mAb’s have been well established in the clinic for their specificity, pharmacokinetics parameters, and engineering ability [2]. The selection of the fluorophore that is linked to the antibody is critical as the dye molecule itself changes the in-vivo behavior of the entire conjugate and the final pharmacokinetic properties of the antibody-dye conjugate [3]. The biochemical behavior of the fluorophore molecule affects the overall stability, fluorescence intensity, target avidity, and biodistribution of the probe which impacts the clinical applicability and success of the molecule [4].

Near-infrared (NIR) heptamethine cyanine fluorophores are preferred agents for tumor visualization and FGS because they operate in a spectral window (700–900 nm) where photons exhibit favorable properties including greater tissue depth penetration, reduced light scattering, and low endogenous tissue autofluorescence [5]. However, these dyes are prone to aggregation due to their planar, hydrophobic, and π-conjugated structures that favor π–π stacking and H-/J-aggregation in aqueous environments [6]. To mitigate the impact of the fluorophore on probe pharmacokinetics, several strategies have been explored. Modification of the conjugate architecture by either site-specific labeling or distancing the dye using hydrophilic flexible polyethylene gylcol (PEG) linkers have been explored [7, 8]. Charge modification by incorporation of zwitterionic or polar functional groups on fluorophores has been shown to reduce aggregation and improve hydrophilicity [9–12]. These modifications have facilitated the development of optical imaging agents with enhanced in-vivo performance and translational potential.

We have previously evaluated a humanized mAb anti-CEA antibody (hT84.66-M5A monoclonal mAb linked to the NIR fluorophore, IRDye800CW (LICORbio, Lincoln, NE) for enhanced visualization of GI cancers [13–19]. The IRDye800CW is a sulfonated heptamethine cyanine that is widely used due to its commercial availability and straightforward conjugation. Its use with other antibodies targeting the epidermal growth factor receptor (EGFR) [20–22] and vascular endothelial growth factor (VEGF) [23] have advanced to clinical trials, indicating its translational relevance.

While these early clinical studies demonstrate promising proof of concept in target labeling, there remains significant opportunities to further optimize the fluorophore to obtain a brighter signal and higher contrast at the target of interest. To improve the signal, we increased the degree of labeling (DOL), but this resulted in unfavorably rapid blood clearance of the fluorescent probe [8]. This is a finding that is consistent with other reports that a maximal DOL of 1.0–1.5 is optimal for such conjugates [24, 25]. To address this limitation, we developed the "M5A-sidewinder," a construct using site-specific conjugation and a polyethylene glycol (PEG) linker to achieve a high DOL (average of 7 dyes per antibody) while maintaining the pharmacokinetics of the parental antibody [8]. Although this conjugate yielded a 4.3-fold signal increase at the tumor, its multi-step synthesis presents a barrier to large-scale production and clinical translation.

Given the manufacturing complexity of the PEGylated conjugate and the described limitations of IRDye800CW, a more direct solution is to improve the intrinsic chemical properties of the fluorophore. We investigated a class of charge-neutral C4'-O-alkyl heptamethine cyanine derivatives, known as Fredricks Near Infrared-Tag dyes (FNIR), which were designed specifically to reduce aggregation and non-specific binding [26, 27]. We hypothesized that these dyes would enable the M5A antibody to achieve a higher effective brightness and tumor-to-background contrast without compromising its pharmacokinetic profile. To test this, the present study assesses the impact of these novel dyes on the optical properties, pharmacokinetics, and biodistribution of the M5A mAb.

## Materials/Methods

### Fluorophore Synthesis and Characterization

The NHS-FNIR-Tag-766 dye was synthesized as described previously [26]. The NHS-FNIR-Tag-804 dye was synthesized as described in the Supplemental Document. Both dyes were characterized as described previously [26]. In brief, molar absorption coefficients (ε) were determined in PBS (pH 7.4) using Beer’s law. Absolute quantum yields (ΦF) were measured using a Quantaurus-QY spectrometer (Hamamatsu, model C11374).

### Antibody-Fluorophore Dye Conjugation

Three near-infrared dyes were used for these studies: (1) IRDye800CW NHS-Ester (excitation 775 nm, emission 796 nm) (LI-CORbio), (2) NHS-FNIR-Tag-766 (excitation 776 nm, emission 788 nm) prepared as described previously [26, 27] and (3) NHS-FNIR-Tag-804 (excitation 804 nm, emission 830 nm) see Supplemental Information for synthesis. The dyes were conjugated to the humanized anti-CEA hT84.66-M5A (M5A) mAb [28]. Conjugation was performed at a 10–15-fold molar excess of dye to antibody. Fresh dye was formulated with a concentration of 10 mg/ml prior to conjugation. The IRDye800CW solution was formulated using Ultra trace water, elemental analysis grade (Fisher Chemical, Pittsburgh, PA). The NHS-FNIR-Tag-766 and NHS-FNIR-Tag-804 were formulated in 10 mM DMSO (Protide Pharmaceuticals: Crystal Lake, IL). Conjugation buffer for the NHS-IRDye800CW was 20 mM sodium phosphate buffer solution (pH 8.5) and conjugation buffer for the NHS-FNIR-Tag-766/NHS-FNIR-Tag-804 was 250 mM phosphate buffer (pH 8.5). The reaction was incubated for 2 h at room temperature on a shaker in the dark. The resulting conjugates were called: M5A-IR800CW, M5A-FNIR-766, M5A-FNIR-804.

### Purification and Characterization of Spectral Properties

The antibody dye conjugates were purified by diafiltration using an Amicon ultrafiltration stirred cell (Millipore Sigma, Burlington, MA) with a 30 kDa molecular weight cut-off membrane. After buffer exchange of 15–20 diavolumes of PBS, the antibody was recovered, and sterile filtered. The protein concentration and DOL were calculated by absorbance spectrophotometry using established equations for the free dye (Eqs. 1 and 2 below). Antibody aggregation and the presence of free dye were analyzed by High-Performance Liquid Chromatography-Size exclusion chromatography (HPLC-SEC) using a Superdex 200 column (Cytiva, Marlborough, MA). The NGC chromatography system (Biorad, Hercules, CA) monitored absorbance at wavelengths of 280 nm and 700 nm. This was equipped with an external Jasco FP4020 fluorescent detector (Jasco Inc., Easton, MD, USA) set to the peak excitation and emission wavelengths for each free dye. The absorption scan of antibody-dye conjugates in PBS was performed using the GENESYS 150 Vis/UV–Vis Spectrophotometers (ThermoFisher Scientific, Waltham, MA). These scans are used to qualitatively evaluate the formation of H-aggregates, which are indicated by an absorption shoulder or peak near 700 nm.

The DOL was calculated using Eq. 1:

1 $$Digree\; of \;Labeling = \frac{{A}_{(Dye max)} \times {\varepsilon }_{(Ab)}}{{\varepsilon }_{(Dye)} \times [{A}_{(280)} - \;Correction\; Factor \times {A}_{(Dye max)}]}$$

The antibody concentration (mg/mL) was determined using Eq. 2:

2 $$Concentration \;(mg/ml) = Dilution\; Factor\; \times \frac{MA_{(Ab)} \;[{A}{(280)} -Correction\; Factor \times {A}{(Dye max)}]}{{\varepsilon }{(Dye)}}$$

### SEC-Based Binding Assay

To confirm conjugate purity and retention of antigen binding, SEC was performed on a Superdex 200 column. Conjugates (10 µg) were analyzed alone or after a 30 min incubation with soluble CEA protein (50 µg) at 37 °C. The elution profiles were monitored by absorbance at 280 nm.

### In-Vitro Studies

Serial dilutions of M5A-fluorophore conjugates in PBS were performed. Protein concentrations were as follows: 1.5 mg/mL, 1 mg/mL, 0.5 mg/mL, 0.25 mg/mL, 0.125 mg/mL, 0.0625 mg/mL, 0.03125 mg/mL, 0.0156 mg/mL. 100μL of the samples were placed in PCR tubes and imaged under the 800 nm channel using the LICOR Pearl Trilogy Small Animal Imager (LICOR Bio, Lincoln, NE).

### Cell Culture

The LS174T human colon cancer cell line expressing firefly luciferase (LS174T-LUC +) was used for this study. Cells were cultured in Dulbecco's Modified Eagle Medium (DMEM) media supplemented with Fetal Bovine Serum (FBS). The cells were passaged at least twice and were 90% confluent prior to in-vivo injection.

### In-Vivo Studies

All animal studies were performed in accordance with regulations set by the City of Hope and University of California, San Diego Animal Care Program under an approved IACUC protocol.

#### Subcutaneous Tumor Mouse Models

Nude mice received subcutaneous injection of 1 × 10^6^ LS174T-Luc + cells to establish the subcutaneous xenograft mouse models. Tumors were followed until they measured approximately 100-200mm^3^.

#### Orthotopic Mouse Models of Colorectal Cancer

Subcutaneous tumors were harvested and sliced into 1-2mm^3^ fragments. Mice were anesthetized per IACUC protocol. A lower abdominal laparotomy was made on recipient nude mice The sigmoid colon was extracted through the laparotomy. A tumor fragment was sutured onto the sigmoid colon using 8–0 nylon sutures to create a surgical orthotopic model of colorectal cancer [29]. The sigmoid colon was replaced into the abdominal cavity and the laparotomy was closed with 6–0 nylon sutures. Mice were monitored and pain medication was provided per IACUC protocol.

#### Antibody Fluorophore Conjugate Injection

Mice were fed a low fluorophore diet 5 days prior to injection of the antibody-fluorophore conjugate. Each mouse was injected with 50 ug of M5A-fluorophore conjugates intravenously via the tail vein.

#### Fluorescence Imaging

Mice with subcutaneous tumors were serially imaged at the following time points: 0 h, 4 h, 24 h, 48 h, 72 h, and 96 h. The number of mice analyzed per group was as follows: n = 8 for M5A-IR800CW at all time points; for both the M5A-FNIR-766 and M5A-FNIR-804 groups, n = 4 at 0–4 h, n = 9 at 24–48 h, and n = 6 at 72–96 h. At the conclusion of the non-invasive imaging studies (At 96 h), n = 6-7mice per group were euthanized for necropsy and optical biodistribution of fluorescence. Mice with orthotopic colorectal tumors (n = 2 M5A-IR800CW, n = 3 M5A-FNIR-766 and n = 3 M5A-FNIR-804) were imaged at 48 h after intravenous probe injection. Mice were euthanized and imaged non-invasively, after laparotomy, and necropsy. In-vivo imaging was performed using the Pearl Trilogy small animal imaging system (LICORbio) using the 800 nm channel with peak excitation at 785 nm and emission at 820 nm (and if applicable, bioluminescence channel) under isoflurane anesthesia [30]. The distance from the imaging lens to the tissue surface was standardized across all mice. The Pearl Trilogy imaging system has a fixed-position specimen stage and camera. All acquired images in the series were linked during analysis; any adjustments to brightness and contrast were applied globally across the entire dataset across groups. Mean fluorescence intensity (MFI) over the tumor and adjacent normal organ was measured using the ImageStudio software (LICORbio). The tumor-to-background ratio (TBR) was calculated for each animal.

#### Statistical Analysis

Quantitative data are presented as mean ± standard error of the mean (SEM). Statistical comparisons between the groups of conjugates was performed using a one-way analysis of variance (ANOVA) followed by Tukey’s post-hoc test for multiple comparisons. A P-value < 0.05 was considered statistically significant. All statistical analyses were performed using GraphPad Prism (Version X, GraphPad Software, San Diego, CA).

### Immunohistochemistry

At the conclusion of macroscopic imaging, tissue samples were collected. Samples were placed in 4% paraformaldehyde and placed into formalin-fixed paraffin embedded blocks. The blocks were serially sectioned at 4–5 um thickness and processed for standard hematoxylin and eosin (H&E) and immunohistochemistry. The slides were deparaffinized, rehydrated and incubated with endogenous peroxydase activity inhibitor and antigen retrieval solution. The slides were incubated with biotinylated goat anti-human IgG antibody (H + L, Cat#: BA-3000) following by OmniMap anti-goat-HRP (Cat#: 760–4647) incubation. The stains were visualized by DISCOVERY ChromoMap DAB Kit (Ventana) and counterstained with hematoxylin and coverslipped. All IHC stains were performed on a Ventana Discovery Ultra IHC automated stainer (Ventana Medical Systems, Roche Diagnostics, Indianapolis, USA). Whole slide images were acquired with Zeiss Observer II Light microscope (Leica Microsystems, Buffalo Grove, IL). Fluorescence microscopy was performed on the unstained slide using the BXZ-710 microscope (Keyence America, Itasca, IL) with the ICG cube (Chroma Technology Corp, Bellows Falls, VT).

## Results

**Fig. 1:**
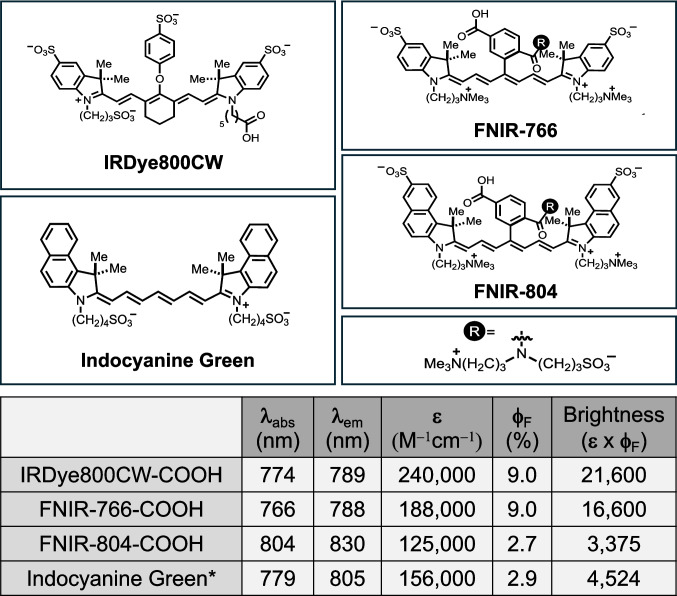
Chemical structures and properties of the fluorescent dyes used in this study in PBS (**l**_abs_—Wavelength absorption, **l**_em_—Wavelength emission, **e** – Extinction coefficient, **f**_F_—Quantum yield). Brightness = (**e)** x (**f**_F_). Values were determined experimentally in PBS unless otherwise noted (*Indocyanine Green spectral properties in water as a reference. Water is the recommended solvent for ICG). The values shown for the previously reported probes, FNIR-Tag-766, IR-800CW and ICG, were reported in references, [26, 31], respectively

The chemical structures and properties of the NIR fluorescent dyes used in the study are summarized in Fig. 1. The FNIR-766 fluorophore has similar spectral properties to the commercially available IRDye800CW. While its quantum yield is comparable (8.7% FNIR-766 vs. 9.0% IRDye800CW), its molar extinction coefficient is approximately 22% lower (188,000 vs. 242,000 M^−1^ cm^−1^). The FNIR-804 dye was designed to have comparable fluorescence properties to Indocyanine Green (ICG). Its peak excitation and emission wavelengths as well as its extinction coefficient and quantum yield are comparable to that of ICG in water [31].

The humanized anti-CEA M5A mAb was conjugated to three near-infrared dyes using amine chemistry to produce the following: 1) IRDye800CW NHS-ester (excitation 775 nm, emission 796 nm) 2) NHS-FNIR-Tag-766 (excitation 776 nm, emission 788 nm) and 3) NHS-FNIR-Tag-804. The conjugates were called M5A-IR800CW, M5A-FNIR-766, and M5A-FNIR-804 respectively. The antibody dye conjugates were purified by diafiltration and their antibody: dye DOL was calculated by absorbance. The DOL for M5A-IR800CW, M5A-FNIR-766, and M5A-FNIR-804 were 3.2, 2.33 and 3.8 respectively.

**Fig. 2:**
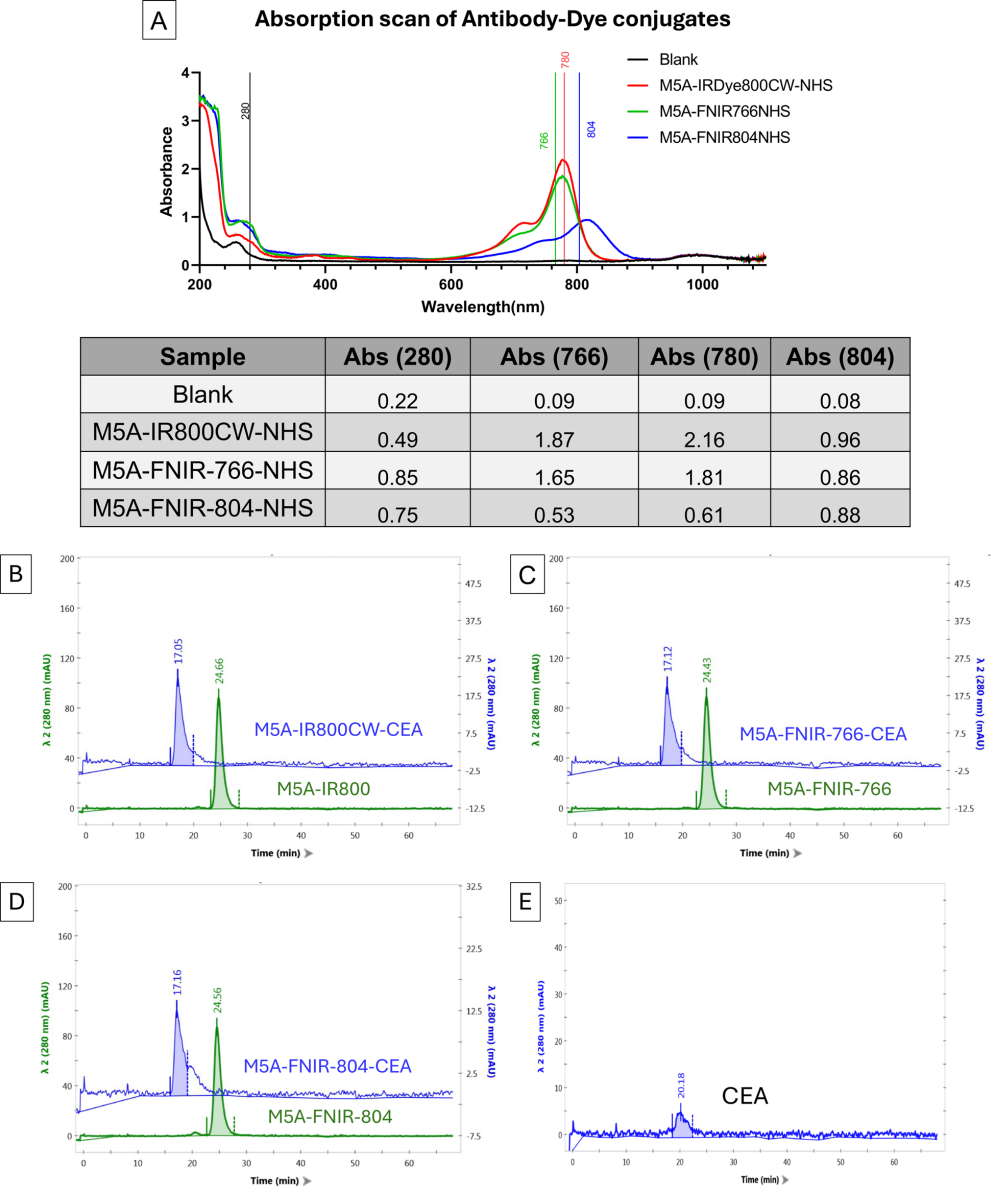
The absorption scan of antibody-dye conjugates in PBS was performed using the GENESYS 150 Vis/UV–Vis Spectrophotometer (ThermoFisher Scientific, Waltham, MA) which monitored absorbance at wavelengths of 280 and 700 (**A**). It is equipped with an external FP4020 fluorescent detector (Jasco Inc., Easton, MD, USA) set to the peak excitation and emission wavelengths for each free dye. There is an H-aggregate peak at around 700 nm for IR800CW which is decreased with the FNIR dyes. Conjugated antibodies (10 µg) were incubated with CEA and analyzed on a Superdex 200 column. Green traces: M5A–dye conjugates alone; blue traces: post-incubation with the CEA antigen. Elution profiles of the conjugates alone (green traces) show that (**B**) M5A-IR800CW, (**C**) M5A-FNIR-766, and (**D**) M5A-FNIR-804 all exist predominantly as a single monomeric peak without aggregation. Post-incubation with the CEA antigen show retention shifts, indicating retention of antigen binding affinity. (**E**) Elution profile of CEA antigen alone

The absorption scan of antibody-dye conjugates in PBS is shown in Fig. 2A. There is an H-aggregate peak at approximately 700 nm for M5A-IR800CW which is decreased with the M5A-FNIR dyes. SEC analysis confirmed that all three conjugates were pure and predominantly monomeric prior to antigen exposure (Fig. 2B-D, green traces). To confirm that high DOL did not impede antigen binding, we performed a SEC-based antigen binding assay. Following incubation with CEA, all three conjugates showed a clear shift in their elution profiles, with the appearance of a high-molecular-weight peak corresponding to the antibody-antigen complex (Fig. 2B-D, blue traces). This confirmed that the conjugates retain their ability to specifically bind their target.

**Fig. 3:**
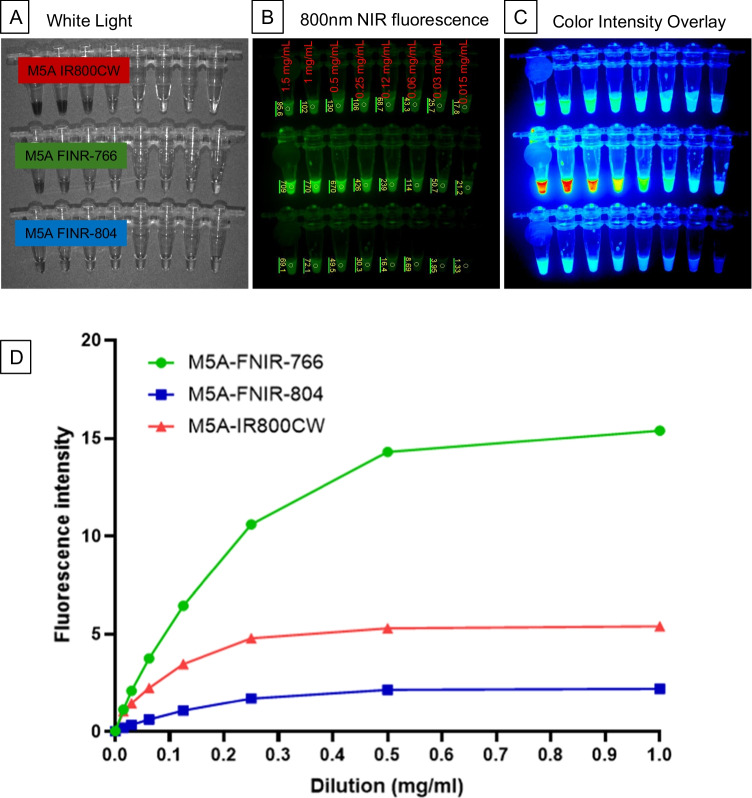
In-vitro imaging of serial dilutions of the antibody fluorophore conjugates in PBS using the LICOR-Pearl Near-Infrared Small Animal Imager showed the brightest fluorescence signal from M5A-FNIR-766 conjugate at all concentrations (**A**) White light image, (**B**) NIR fluorescence 800 nm image, (**C**) Color-Intensity Overlay. Quantification of fluorescence (**D**) demonstrated nearly an increased brightness observed from M5A-FNIR-766 vs. M5A-IR800CW at all concentrations. M5A-FNIR-804 had decreased fluorescence intensity across all concentrations.

To evaluate the in-vitro fluorescence signal intensity of the antibody-dye conjugates, serial dilutions of the antibody fluorophore conjugates in PBS were imaged using the Pearl Near-Infrared Small Animal Imager. The M5A-FNIR-766 showed the brightest fluorescence signal at all concentrations (Fig. 3A) White light image, (Fig. 3B) NIR fluorescence 800 nm image, (Fig. 3C) Color-Intensity Overlay. Quantification of fluorescence (Fig. 3D) demonstrated nearly an increased brightness observed from M5A-FNIR-766 vs. M5A-IR800CW at all concentrations. M5A-FNIR-804 had decreased fluorescence intensity across all concentrations.

**Fig. 4:**
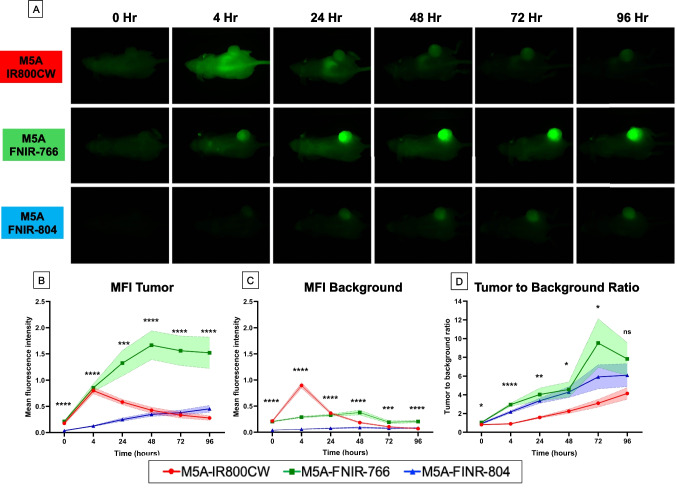
In-vivo imaging of LS174T-Luc + subcutaneous human colorectal cancer bearing mice after intravenous injection with M5A-fluorophore conjugates using the LICOR Pearl Trilogy Small Animal Imager. Serial imaging using the 800 nm channel on the imager (**A**) was performed. Fluorescence intensity maps were processed all groups as linked series. The mean fluorescence signal of the tumor (**B**) and adjacent background tissue (**C**) was measured. The TBR was calculated by dividing the MFI at the tumor by the MFI at the background (**D**). Data are presented as mean ± SEM. Statistical analysis was performed by one-way ANOVA with Tukey’s post-hoc multiple comparison test (Supplemental data). Asterisks denote levels of statistical significance: ns = not significant (*p* ≥ 0.05); * *p* < 0.05; ** *p* < 0.01; *** *p* < 0.001; **** *p* < 0.0001. Sample size: n = 4–9 mice per group per time point

In-vivo imaging of the LS174T-Luc + subcutaneous human colorectal cancer bearing mice was performed after intravenous injection with M5A-fluorophore conjugates using the LICOR Pearl Trilogy Small Animal Imager (Fig. 4A) using the 800 nm channel on the imager. The MFI over the tumor and adjacent tissue was quantified using the LICOR Image studio software (Fig. 4B and C). Both the M5A-IR800CW and M5A-FNIR-766 conjugates had comparable MFI over the tumor at 0 and 4 h (ns, p > 0.2), while M5A-FNIR-804 exhibited significantly lower signal than both conjugates (**** p < 0.0001, Tukey post-hoc; Supplemental Table 1). At all the time points, the M5A-FNIR-766 conjugate had the highest MFI over the tumor. By 24 h, M5A-FNIR-766 demonstrated a significantly higher tumor MFI than M5A-IR800CW (p = 0.0057, **). The peak MFI for the M5A-IR800CW conjugate was observed at 4 h and the fluorescence signal over the tumor decreased thereafter. In comparison, the fluorescence intensity over the tumor of the M5A-FNIR-766 peaked at 48 h and remaining significantly higher than both M5A-IR800CW and M5A-FNIR-804 from 24 h, until 96 h (**–**** p < 0.001–0.0001), the endpoint of the experiment. At 48 h, M5A-FNIR-766 demonstrated a fourfold higher tumor MFI compared to M5A-IR800CW and threefold higher tumor MFI compared to M5A-FNIR-804. The M5A-FNIR-804 continued to have a steady increase in MFI over the time studied.

Analysis of the background signal indicated that both the M5A-IR800CW and M5A-FNIR-766 conjugates had some nonspecific background signal observable over the entire mouse that decreased at later timepoints. The M5A-FNIR-804 conjugate however, had the lowest background signal at all time points (**** p < 0.0001).

The TBR was calculated as a ratio of the tumor signal divided by the background signal (Fig. 4D). The steady increase in TBR over time of all conjugates was driven by a faster washout of the background signal compared to the fluorescence accumulation at the tumor. M5A-FNIR-766 had a significantly higher TBR than M5A-IR800CW at 4 h (**** p < 0.0001), 24 h (** p = 0.0024), 48 h (* p = 0.0357), and 72 h (* p = 0.0193). With a lower MFI at the tumor, but a much lower background MFI, the M5A-FNIR-804 resulted in intermediate TBR at all time points. M5A- IR800CW had the lowest TBR at all time points.

**Fig. 5:**
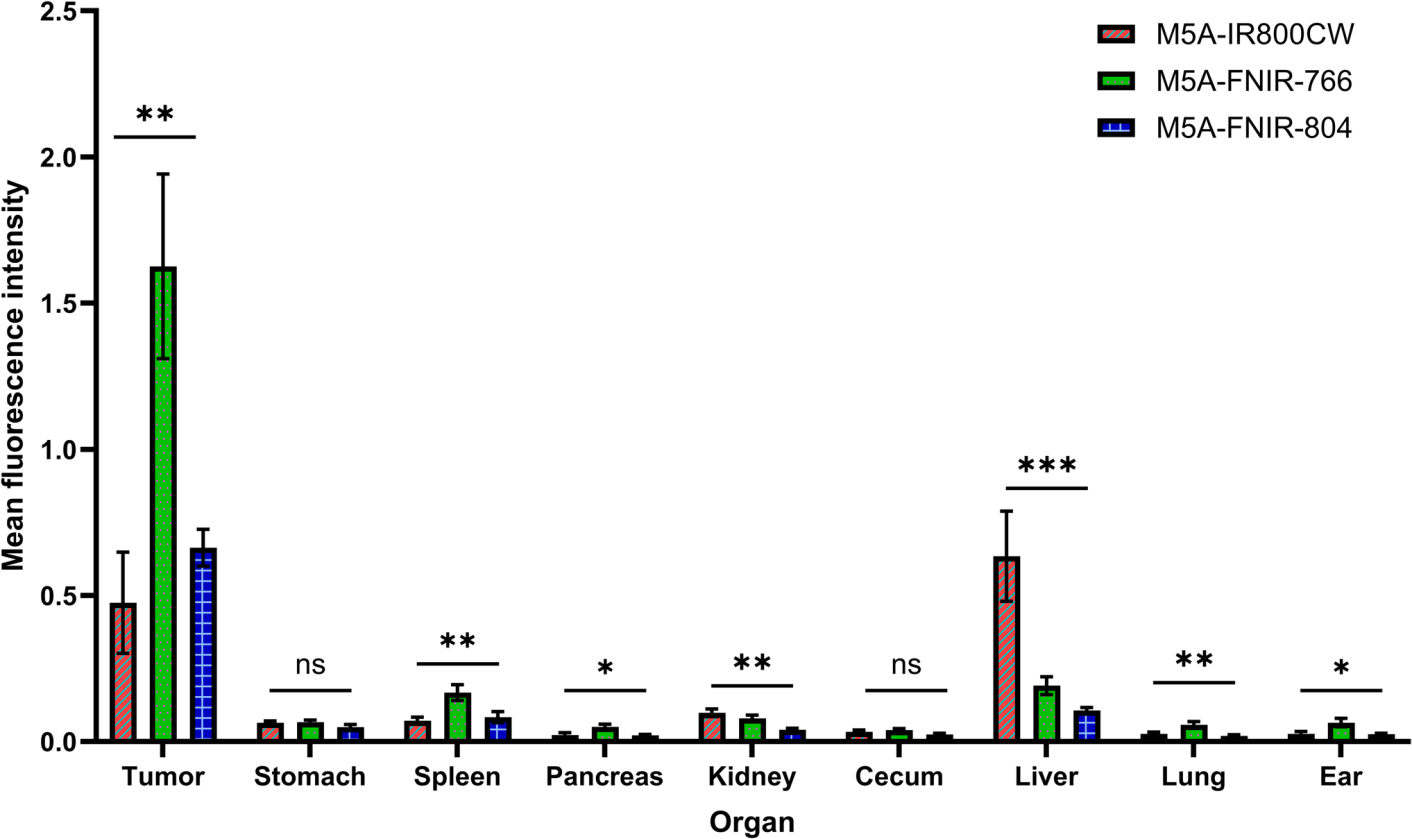
Optical biodistribution was performed on necropsy specimen at 96 h using the LICOR Pearl Trilogy. Quantification of fluorescence intensity across major organs revealed that M5A-FNIR-766 exhibited the highest tumor signal compared to both M5A-IR800CW (p = 0.0047, **) and M5A-FNIR-804 (*p* = 0.0129, ***), while maintaining limited off-target accumulation. FNIR conjugates demonstrated significantly reduced hepatic uptake relative to M5A-IR800CW (M5A-FNIR-766 vs M5A-IR800CW *p *= 0.0040, **; M5A-FNIR-804 vs M5A-IR800CW *p* = 0.0009, ***). Data are shown as mean ± SEM. Statistical analysis was performed using a one-way ANOVA with Tukey’s post-hoc multiple comparison test. Asterisks denote significance: ns = not significant (*p* ≥ 0.05); * *p* < 0.05; ** *p* < 0.01; *** *p* < 0.001; **** *p* < 0.0001. n = 6–7 mice per group

An optical biodistribution was performed by performing a necropsy and measuring the ex-vivo fluorescence signal over the following specimens: tumor, stomach, spleen, pancreas, kidney, cecum (colon), liver, lung, ear tag (skin) (Fig. 5). Consistent with the in-vivo data, the fluorescence signal at the tumor with the M5A-FNIR-766 probe demonstrated the highest MFI, followed by the M5A-IR800CW and the M5A-FNIR-804. Quantification of fluorescence demonstrated that the FNIR conjugates had comparable to decreased fluorescence signal at all major organs except the spleen and pancreas. The M5A-IR800CW conjugate had the highest liver signal which was threefold greater than that of the FNIR conjugates.

**Fig. 6:**
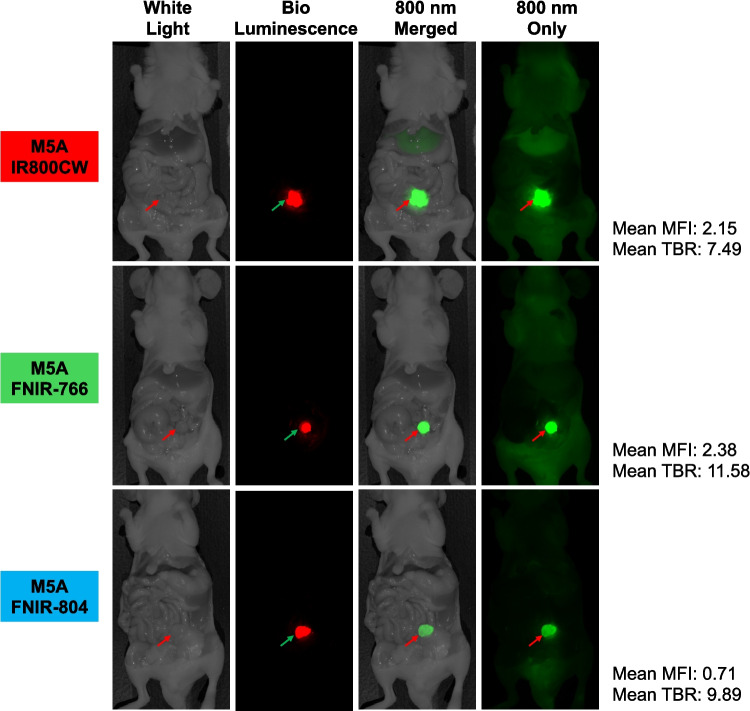
Mice bearing LS174T-Luc + human colorectal cancers orthotopically implanted over the sigmoid colon were administered M5A-fluorophore conjugates and sacrificed at 48 h. Fluorescence signals were highest with the M5A-FNIR-766 probe, followed by the M5A-IR800CW, and the M5A-FNIR-804. The M5A-FNIR-766 conjugate demonstrated the highest TBR in the orthotopic models, followed by the M5A-FNIR-804 conjugate, while the lowest TBR was observed in the M5A-IR800CW conjugate. There was detectable liver fluorescence with the M5A-IR800CW probe in the orthotopic model which is not present with the FNIR conjugates

**Fig. 7:**
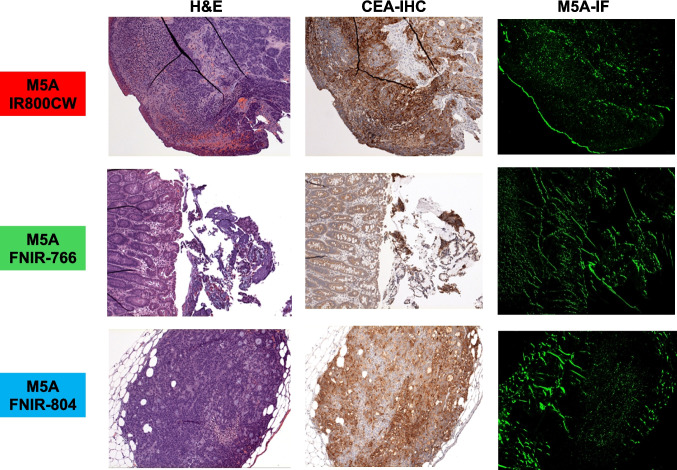
Immunohistochemistry and immunofluorescence imaging showed concordance of CEA expression with the fluorescence signal at a microscopic level. Light microscopy was performed using the Zeiss Observer II Light microscope at the 10× objective and fluorescence microscopy was performed using the Keyence BXZ microscope using the ICG cube

Mice bearing LS174T-Luc + human colorectal cancers orthotopically implanted over the sigmoid colon were administered M5A-fluorophore conjugates and sacrificed at 48 h (Fig. 6). Fluorescence signals over the tumor were highest with the M5A-FNIR-766 probe, followed by the M5A-IR800CW, and the M5A-FNIR-804. Consistent with the data trend observed in the subcutaneous tumor models, the M5A-FNIR-766 conjugate demonstrated the highest TBR in the orthotopic models, followed by the M5A-FNIR-804 conjugate, while the lowest TBR was observed in the M5A-IR800CW conjugate. Pronounced liver fluorescence was observed with the M5A-IR800CW probe in the orthotopic tumor model which can present a false-positive signal. Immunohistochemistry and immunofluorescence imaging showed concordance of CEA expression with the fluorescence signal at a microscopic level (Fig. 7).

## Discussion

The use of modified charge-neutral heptamethine cyanines (FNIR) fluorophores linked to the humanized anti-CEA antibody M5A demonstrated both improved fluorescence intensity and contrast compared to the commercial NIR fluorophore IRDye800CW. The degree of enhancement in fluorescence represents a significant advancement beyond what would be predicted from the recorded spectral properties of the dyes. Based on the respective extinction coefficient and quantum yield of the dyes (Fig. 1), the calculated brightness of the IR800CW dye is expected to be higher than that of both heptamethine FNIR dyes, yet our data reveals a strikingly different in-vivo performance profile.

Using serial dilutions of the antibody-dye conjugates, the M5A-FNIR-766 conjugate demonstrated the highest MFI in phantom studies (Fig. 3). This improved optical performance was also observed *in vivo*: M5A-FNIR-766 demonstrated a significant and sustained increase in tumor-associated fluorescence compared with both M5A-IR800CW and M5A-FNIR-804 (Fig. 4). Quantitative analysis confirmed that differences between conjugates became statistically significant by 24 h post-injection, when M5A-FNIR-766 showed markedly higher tumor MFI than M5A-IR800CW (p = 0.0057*) and M5A-FNIR-804 (p < 0.0001) (Supplemental Table 1). This difference continued to increase through 48–96 h, during which M5A-FNIR-766 achieved approximately a four-fold increase in tumor MFI relative to M5A-IR800CW and more than three-fold higher intensity than M5A-FNIR-804. The previously studied panitumumab-FNIR-766 conjugate in mouse models of breast cancer which also showed an improvement with the FNIR-766 dye [26]. In these published studies with panitumumab, there was a 2.5-fold improvement, while in the present experiments we observed a fourfold improvement in fluorescence intensity over the tumor. With increasing MFI over the tumor, an increase in the background signal is expected as this is a phenomena observed with other fluorescent dyes [24, 25]. M5A-FNIR-766 displayed significantly lower background fluorescence than M5A-IR800CW beginning at 48 h (p = 0.0004), and comparable background thereafter, indicating more efficient clearance from non-target tissues. This led to an improvement in the TBR of the M5A-FNIR-766 probe which was superior to the other two probes at multiple time points (4 h – 72 h, p < 0.05). Despite having a lower fluorescence signal over the tumor, the M5A-FNIR-804 probe had the lowest background signal at all time points (**** p < 0.0001) which unexpectedly resulted in intermediate TBR values. The performance gap between theoretical brightness calculations and actual in-vivo performance underscores the importance of charge distribution and aggregation properties in determining the in-vivo effectiveness of fluorophore-antibody conjugates [32].

The biodistribution profile of the FNIR conjugates represents another significant translational advantage. Optical biodistribution studies of the necropsy samples confirmed the superior performance of the FNIR-766 dye, which had the highest signals over the tumor while maintaining minimal uptake in other organs. Quantitative post-hoc analysis showed significantly greater tumor accumulation for M5A-FNIR-766 compared with M5A-IR800CW (p = 0.0047, ***) and M5A-FNIR-804 (p = 0.0129, ***). The substantial reduction in liver signal with both FNIR conjugates (threefold lower than M5A-IR800CW) addresses a major limitation in current fluorescence-guided surgery approaches during intra-abdominal procedures; high fluorescence signals in adjacent hepatobiliary anatomy can confound visualization of the true target. The non-specific liver signal with the M5A-IR800CW probe is above that of standard antibody accumulation in the liver and is likely driven by the hydrophobicity of the IR800CW dye [3, 11]. The placement of quaternary amines at the central carbon and tri-ethylene gylcol groups on the indolenine position resulted in dyes with decreased liver uptake [26, 33]. Both M5A-FNIR-766 and M5A-FNIR-804 demonstrated significantly lower liver fluorescence than M5A-IR800CW (M5A-FNIR-766 vs M5A-IR800CW p = 0.0040, **; M5A-FNIR-804 vs M5A-IR800CW p = 0.0009, ***). This enhancement in signal intensity, coupled with lower background signal (especially at early time points), offers significant clinical advantages for surgical visualization.

Given that the adjacent skin is not a reliable background signal for intra-abdominal processes, we compared these dyes in an orthotopic model of human colorectal cancer [34]. By evaluating probe delivery in the native anatomic environment with true physiological background signals from adjacent bowel tissue, we confirmed that the performance advantages observed in subcutaneous studies translate to clinically relevant models. In this model, we observed concordant trends in MFI and TBR’s at 48 h that were observed in the subcutaneous model (Fig. 5). The bright non-specific liver signal observed in the subcutaneous necropsy specimens of mice that received M5A-IR800CW at 96 h was also observed in-situ in the orthotopic model, but not with the M5A-FNIR dyes. While the orthotopic model provides a more clinically relevant tumor microenvironment and surgical simulation, the technical challenges of developing orthotopic models limited our initial evaluation to a small sample size (n = 2–3 mice per group). These promising results warrant confirmation in larger future studies.

This present work seeks to evaluate a series of novel, charge-balanced modified heptamethine fluorophores designed to overcome the classical limitations of conventional cyanines dyes such as conventional cyanine dyes, such as chemical instability, non-specific binding, and self-aggregation leading to fluorescence quenching. Rational fluorophore design is a potential solution to addresses these challenges. Charge-balanced, or zwitterionic, structures that reduce non-specific protein binding and promote rapid renal clearance have been evaluated [10]. Increasing the asymmetry of charge density within the fluorophore and increasing its hydrophilicty is another approach [12]. Other groups have used steric shielding to to physically block the polyene core from interacting with itself or off-target biomolecules [9]. Our approach of using a net neutral intramolecular charge balance to disrupt the π-stacking responsible for H-aggregation permits the use of higher degrees of labeling (DOL) without the typical penalty to quantum yield, resulting in agents with greater per-molecule brightness and superior in-vivo performance. These strategies of rational dye development represent a significant advancement over previous extrinsic strategies, such as site-specific conjugation or PEGylation to shield the hydrophobic fluorophore, which add complexity and cost to manufacturing [8]. Achieving superior fluorescent probe performance through rational dye development can simplify GMP manufacturing using standard antibody-dye conjugation protocols with established regulatory approval. The FNIR-766 dye that exemplifies this strategy and provide both an increased fluorescence signal and improved contrast.

The FNIR-804 dye could have potential advantages when a higher contrast is desired at the cost of signal strength. It is important to note that the fluorescence imaging performed in these experiments utilized the Pearl Trilogy small animal imager with the “800 nm” channel which has a peak excitation wavelength of 785 nm and an emission filter of 820 nm which are optimized for the peak spectra of fluorophores such as IR800CW dye. While this instrument is well-suited for M5A-IR800CW and M5A-FNIR-766, its emission filter is suboptimal for detecting the peak emission of FNIR-804 (~ 830 nm), a factor that contributed to its lower detected signal in our experiments. This is a strategic consideration, as FNIR-804 was designed to be compatible with the large installed base of clinical imaging devices developed for ICG which are already present in surgical suites worldwide. This compatibility would eliminate the need for specialized imaging equipment and reduce barriers to clinical adoption.

These findings have significant implications for accelerating the clinical translation of fluorescence-guided surgery. The improvement in tumor-to-background contrast without compromising the favorable pharmacokinetics of the antibody backbone enables the use of lower doses while maintaining imaging performance. This enhanced performance profile could potentially translate to improved precision in detection of smaller tumor deposits. Future directions will focus on optimizing the DOL for the M5A-FNIR-766 conjugate, as these modified dyes may tolerate even higher labeling density without aggregation compared to traditional cyanine dyes. Optimization, coupled with the already improved performance, would enable rapid advancement to clinical testing. The standardized conjugation chemistry will facilitate good manufacturing practice (GMP) manufacturing, further accelerating the pathway to first-in-human studies.

This present work represents a significant advancement in fluorophore design that directly addresses key barriers to clinical translation. The M5A-FNIR-766 conjugate demonstrates clear advantages in terms of tumor signal intensity, background reduction, and biodistribution that not only enhance surgical visualization. The amine conjugation strategy addresses regulatory concerns and manufacturing challenges that can delay clinical implementation. The straightforward conjugation approach, compatibility with existing imaging systems, and favorable pharmacological properties make these agents ideal for accelerated clinical translation.

## Supplementary Information

Below is the link to the electronic supplementary material.

ESM 1 (PPTX 61.2 KB)
