# Supplementary material for: Enhanced Fluorescence of Near-Infrared Anti-CEA Antibodies for Visualizing Colorectal Cancers Using Modified Heptamethine Cyanines
Source: Mol Imaging Biol. 2026 Feb 10;28(2):369–81. doi: 10.1007/s11307-025-02076-3 (PMC13161264; doi:10.1007/s11307-025-02076-3)
Supplement: Supplementary file 1 — (PPTX 61.2 KB) [file 11307_2025_2076_MOESM1_ESM.pptx]

## Slide 1
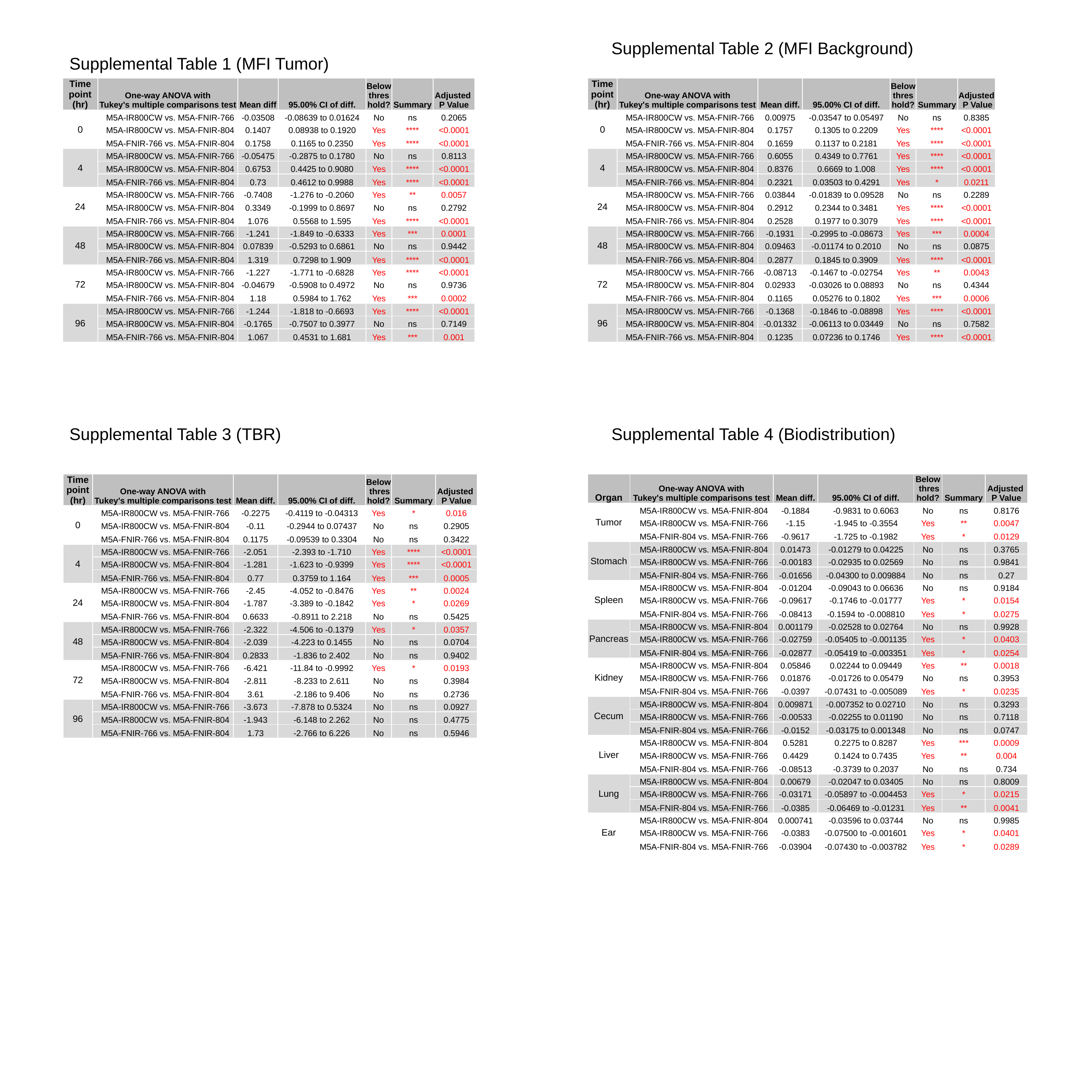

Supplemental Table 2 (MFI Background)
Supplemental Table 1 (MFI Tumor)
| Time point (hr) | One-way ANOVA with Tukey's multiple comparisons test | Mean diff. | 95.00% CI of diff. | Belowthreshold? | Summary | Adjusted P Value |
| --- | --- | --- | --- | --- | --- | --- |
| 0 | M5A-IR800CW vs. M5A-FNIR-766 | 0.00975 | -0.03547 to 0.05497 | No | ns | 0.8385 |
| | M5A-IR800CW vs. M5A-FNIR-804 | 0.1757 | 0.1305 to 0.2209 | Yes | \*\*\*\* | <0.0001 |
| | M5A-FNIR-766 vs. M5A-FNIR-804 | 0.1659 | 0.1137 to 0.2181 | Yes | \*\*\*\* | <0.0001 |
| 4 | M5A-IR800CW vs. M5A-FNIR-766 | 0.6055 | 0.4349 to 0.7761 | Yes | \*\*\*\* | <0.0001 |
| | M5A-IR800CW vs. M5A-FNIR-804 | 0.8376 | 0.6669 to 1.008 | Yes | \*\*\*\* | <0.0001 |
| | M5A-FNIR-766 vs. M5A-FNIR-804 | 0.2321 | 0.03503 to 0.4291 | Yes | \* | 0.0211 |
| 24 | M5A-IR800CW vs. M5A-FNIR-766 | 0.03844 | -0.01839 to 0.09528 | No | ns | 0.2289 |
| | M5A-IR800CW vs. M5A-FNIR-804 | 0.2912 | 0.2344 to 0.3481 | Yes | \*\*\*\* | <0.0001 |
| | M5A-FNIR-766 vs. M5A-FNIR-804 | 0.2528 | 0.1977 to 0.3079 | Yes | \*\*\*\* | <0.0001 |
| 48 | M5A-IR800CW vs. M5A-FNIR-766 | -0.1931 | -0.2995 to -0.08673 | Yes | \*\*\* | 0.0004 |
| | M5A-IR800CW vs. M5A-FNIR-804 | 0.09463 | -0.01174 to 0.2010 | No | ns | 0.0875 |
| | M5A-FNIR-766 vs. M5A-FNIR-804 | 0.2877 | 0.1845 to 0.3909 | Yes | \*\*\*\* | <0.0001 |
| 72 | M5A-IR800CW vs. M5A-FNIR-766 | -0.08713 | -0.1467 to -0.02754 | Yes | \*\* | 0.0043 |
| | M5A-IR800CW vs. M5A-FNIR-804 | 0.02933 | -0.03026 to 0.08893 | No | ns | 0.4344 |
| | M5A-FNIR-766 vs. M5A-FNIR-804 | 0.1165 | 0.05276 to 0.1802 | Yes | \*\*\* | 0.0006 |
| 96 | M5A-IR800CW vs. M5A-FNIR-766 | -0.1368 | -0.1846 to -0.08898 | Yes | \*\*\*\* | <0.0001 |
| | M5A-IR800CW vs. M5A-FNIR-804 | -0.01332 | -0.06113 to 0.03449 | No | ns | 0.7582 |
| | M5A-FNIR-766 vs. M5A-FNIR-804 | 0.1235 | 0.07236 to 0.1746 | Yes | \*\*\*\* | <0.0001 |
| Time point (hr) | One-way ANOVA with Tukey's multiple comparisons test | Mean diff | 95.00% CI of diff. | Belowthreshold? | Summary | Adjusted P Value |
| --- | --- | --- | --- | --- | --- | --- |
| 0 | M5A-IR800CW vs. M5A-FNIR-766 | -0.03508 | -0.08639 to 0.01624 | No | ns | 0.2065 |
| | M5A-IR800CW vs. M5A-FNIR-804 | 0.1407 | 0.08938 to 0.1920 | Yes | \*\*\*\* | <0.0001 |
| | M5A-FNIR-766 vs. M5A-FNIR-804 | 0.1758 | 0.1165 to 0.2350 | Yes | \*\*\*\* | <0.0001 |
| 4 | M5A-IR800CW vs. M5A-FNIR-766 | -0.05475 | -0.2875 to 0.1780 | No | ns | 0.8113 |
| | M5A-IR800CW vs. M5A-FNIR-804 | 0.6753 | 0.4425 to 0.9080 | Yes | \*\*\*\* | <0.0001 |
| | M5A-FNIR-766 vs. M5A-FNIR-804 | 0.73 | 0.4612 to 0.9988 | Yes | \*\*\*\* | <0.0001 |
| 24 | M5A-IR800CW vs. M5A-FNIR-766 | -0.7408 | -1.276 to -0.2060 | Yes | \*\* | 0.0057 |
| | M5A-IR800CW vs. M5A-FNIR-804 | 0.3349 | -0.1999 to 0.8697 | No | ns | 0.2792 |
| | M5A-FNIR-766 vs. M5A-FNIR-804 | 1.076 | 0.5568 to 1.595 | Yes | \*\*\*\* | <0.0001 |
| 48 | M5A-IR800CW vs. M5A-FNIR-766 | -1.241 | -1.849 to -0.6333 | Yes | \*\*\* | 0.0001 |
| | M5A-IR800CW vs. M5A-FNIR-804 | 0.07839 | -0.5293 to 0.6861 | No | ns | 0.9442 |
| | M5A-FNIR-766 vs. M5A-FNIR-804 | 1.319 | 0.7298 to 1.909 | Yes | \*\*\*\* | <0.0001 |
| 72 | M5A-IR800CW vs. M5A-FNIR-766 | -1.227 | -1.771 to -0.6828 | Yes | \*\*\*\* | <0.0001 |
| | M5A-IR800CW vs. M5A-FNIR-804 | -0.04679 | -0.5908 to 0.4972 | No | ns | 0.9736 |
| | M5A-FNIR-766 vs. M5A-FNIR-804 | 1.18 | 0.5984 to 1.762 | Yes | \*\*\* | 0.0002 |
| 96 | M5A-IR800CW vs. M5A-FNIR-766 | -1.244 | -1.818 to -0.6693 | Yes | \*\*\*\* | <0.0001 |
| | M5A-IR800CW vs. M5A-FNIR-804 | -0.1765 | -0.7507 to 0.3977 | No | ns | 0.7149 |
| | M5A-FNIR-766 vs. M5A-FNIR-804 | 1.067 | 0.4531 to 1.681 | Yes | \*\*\* | 0.001 |
Supplemental Table 3 (TBR)
Supplemental Table 4 (Biodistribution)
| Time point (hr) | One-way ANOVA with Tukey's multiple comparisons test | Mean diff. | 95.00% CI of diff. | Below threshold? | Summary | Adjusted P Value |
| --- | --- | --- | --- | --- | --- | --- |
| 0 | M5A-IR800CW vs. M5A-FNIR-766 | -0.2275 | -0.4119 to -0.04313 | Yes | \* | 0.016 |
| | M5A-IR800CW vs. M5A-FNIR-804 | -0.11 | -0.2944 to 0.07437 | No | ns | 0.2905 |
| | M5A-FNIR-766 vs. M5A-FNIR-804 | 0.1175 | -0.09539 to 0.3304 | No | ns | 0.3422 |
| 4 | M5A-IR800CW vs. M5A-FNIR-766 | -2.051 | -2.393 to -1.710 | Yes | \*\*\*\* | <0.0001 |
| | M5A-IR800CW vs. M5A-FNIR-804 | -1.281 | -1.623 to -0.9399 | Yes | \*\*\*\* | <0.0001 |
| | M5A-FNIR-766 vs. M5A-FNIR-804 | 0.77 | 0.3759 to 1.164 | Yes | \*\*\* | 0.0005 |
| 24 | M5A-IR800CW vs. M5A-FNIR-766 | -2.45 | -4.052 to -0.8476 | Yes | \*\* | 0.0024 |
| | M5A-IR800CW vs. M5A-FNIR-804 | -1.787 | -3.389 to -0.1842 | Yes | \* | 0.0269 |
| | M5A-FNIR-766 vs. M5A-FNIR-804 | 0.6633 | -0.8911 to 2.218 | No | ns | 0.5425 |
| 48 | M5A-IR800CW vs. M5A-FNIR-766 | -2.322 | -4.506 to -0.1379 | Yes | \* | 0.0357 |
| | M5A-IR800CW vs. M5A-FNIR-804 | -2.039 | -4.223 to 0.1455 | No | ns | 0.0704 |
| | M5A-FNIR-766 vs. M5A-FNIR-804 | 0.2833 | -1.836 to 2.402 | No | ns | 0.9402 |
| 72 | M5A-IR800CW vs. M5A-FNIR-766 | -6.421 | -11.84 to -0.9992 | Yes | \* | 0.0193 |
| | M5A-IR800CW vs. M5A-FNIR-804 | -2.811 | -8.233 to 2.611 | No | ns | 0.3984 |
| | M5A-FNIR-766 vs. M5A-FNIR-804 | 3.61 | -2.186 to 9.406 | No | ns | 0.2736 |
| 96 | M5A-IR800CW vs. M5A-FNIR-766 | -3.673 | -7.878 to 0.5324 | No | ns | 0.0927 |
| | M5A-IR800CW vs. M5A-FNIR-804 | -1.943 | -6.148 to 2.262 | No | ns | 0.4775 |
| | M5A-FNIR-766 vs. M5A-FNIR-804 | 1.73 | -2.766 to 6.226 | No | ns | 0.5946 |
| Organ | One-way ANOVA with Tukey's multiple comparisons test | Mean diff. | 95.00% CI of diff. | Below threshold? | Summary | Adjusted P Value |
| --- | --- | --- | --- | --- | --- | --- |
| Tumor | M5A-IR800CW vs. M5A-FNIR-804 | -0.1884 | -0.9831 to 0.6063 | No | ns | 0.8176 |
| | M5A-IR800CW vs. M5A-FNIR-766 | -1.15 | -1.945 to -0.3554 | Yes | \*\* | 0.0047 |
| | M5A-FNIR-804 vs. M5A-FNIR-766 | -0.9617 | -1.725 to -0.1982 | Yes | \* | 0.0129 |
| Stomach | M5A-IR800CW vs. M5A-FNIR-804 | 0.01473 | -0.01279 to 0.04225 | No | ns | 0.3765 |
| | M5A-IR800CW vs. M5A-FNIR-766 | -0.00183 | -0.02935 to 0.02569 | No | ns | 0.9841 |
| | M5A-FNIR-804 vs. M5A-FNIR-766 | -0.01656 | -0.04300 to 0.009884 | No | ns | 0.27 |
| Spleen | M5A-IR800CW vs. M5A-FNIR-804 | -0.01204 | -0.09043 to 0.06636 | No | ns | 0.9184 |
| | M5A-IR800CW vs. M5A-FNIR-766 | -0.09617 | -0.1746 to -0.01777 | Yes | \* | 0.0154 |
| | M5A-FNIR-804 vs. M5A-FNIR-766 | -0.08413 | -0.1594 to -0.008810 | Yes | \* | 0.0275 |
| Pancreas | M5A-IR800CW vs. M5A-FNIR-804 | 0.001179 | -0.02528 to 0.02764 | No | ns | 0.9928 |
| | M5A-IR800CW vs. M5A-FNIR-766 | -0.02759 | -0.05405 to -0.001135 | Yes | \* | 0.0403 |
| | M5A-FNIR-804 vs. M5A-FNIR-766 | -0.02877 | -0.05419 to -0.003351 | Yes | \* | 0.0254 |
| Kidney | M5A-IR800CW vs. M5A-FNIR-804 | 0.05846 | 0.02244 to 0.09449 | Yes | \*\* | 0.0018 |
| | M5A-IR800CW vs. M5A-FNIR-766 | 0.01876 | -0.01726 to 0.05479 | No | ns | 0.3953 |
| | M5A-FNIR-804 vs. M5A-FNIR-766 | -0.0397 | -0.07431 to -0.005089 | Yes | \* | 0.0235 |
| Cecum | M5A-IR800CW vs. M5A-FNIR-804 | 0.009871 | -0.007352 to 0.02710 | No | ns | 0.3293 |
| | M5A-IR800CW vs. M5A-FNIR-766 | -0.00533 | -0.02255 to 0.01190 | No | ns | 0.7118 |
| | M5A-FNIR-804 vs. M5A-FNIR-766 | -0.0152 | -0.03175 to 0.001348 | No | ns | 0.0747 |
| Liver | M5A-IR800CW vs. M5A-FNIR-804 | 0.5281 | 0.2275 to 0.8287 | Yes | \*\*\* | 0.0009 |
| | M5A-IR800CW vs. M5A-FNIR-766 | 0.4429 | 0.1424 to 0.7435 | Yes | \*\* | 0.004 |
| | M5A-FNIR-804 vs. M5A-FNIR-766 | -0.08513 | -0.3739 to 0.2037 | No | ns | 0.734 |
| Lung | M5A-IR800CW vs. M5A-FNIR-804 | 0.00679 | -0.02047 to 0.03405 | No | ns | 0.8009 |
| | M5A-IR800CW vs. M5A-FNIR-766 | -0.03171 | -0.05897 to -0.004453 | Yes | \* | 0.0215 |
| | M5A-FNIR-804 vs. M5A-FNIR-766 | -0.0385 | -0.06469 to -0.01231 | Yes | \*\* | 0.0041 |
| Ear | M5A-IR800CW vs. M5A-FNIR-804 | 0.000741 | -0.03596 to 0.03744 | No | ns | 0.9985 |
| | M5A-IR800CW vs. M5A-FNIR-766 | -0.0383 | -0.07500 to -0.001601 | Yes | \* | 0.0401 |
| | M5A-FNIR-804 vs. M5A-FNIR-766 | -0.03904 | -0.07430 to -0.003782 | Yes | \* | 0.0289 |
